# Applications of Ruthenium Complexes Covalently Linked to Nucleic Acid Derivatives

**DOI:** 10.3390/molecules23071515

**Published:** 2018-06-22

**Authors:** Marie Flamme, Emma Clarke, Gilles Gasser, Marcel Hollenstein

**Affiliations:** 1 Laboratory for Inorganic Chemical Biology, Chimie ParisTech, PSL University, F-75005 Paris, France; marie.flamme@pasteur.fr (M.F.); 2167935C@student.gla.ac.uk (E.C.); 2 Laboratory for Bioorganic Chemistry of Nucleic Acids, Department of Structural Biology and Chemistry, Institute Pasteur, CNRS UMR3523, 28, rue du Docteur Roux, 75724 Paris CEDEX 15, France

**Keywords:** ruthenium complexes, oligonucleotides, therapeutic applications, electron transfer, DNA nanomaterials

## Abstract

Oligonucleotides are biopolymers that can be easily modified at various locations. Thereby, the attachment of metal complexes to nucleic acid derivatives has emerged as a common pathway to improve the understanding of biological processes or to steer oligonucleotides towards novel applications such as electron transfer or the construction of nanomaterials. Among the different metal complexes coupled to oligonucleotides, ruthenium complexes, have been extensively studied due to their remarkable properties. The resulting DNA-ruthenium bioconjugates have already demonstrated their potency in numerous applications. Consequently, this review focuses on the recent synthetic methods developed for the preparation of ruthenium complexes covalently linked to oligonucleotides. In addition, the usefulness of such conjugates will be highlighted and their applications from nanotechnologies to therapeutic purposes will be discussed.

## 1. Introduction

The fantastic properties of DNA have made it a tool of choice for numerous applications. Due to its high fidelity of hybridization, selective, and programmable self-assembly properties and ease of synthesis, DNA has indeed emerged as a promising template for the construction of nanostructures [1,2,3,4,5,6]. Its double helical nature, consisting of a rigid, aromatic column of stacked base pairs, allows charge and electron transfer through DNA duplexes [7,8,9]. As carriers of genetic information and blueprints for protein synthesis, DNA and RNA, respectively, have also risen as targets for the inhibition of gene expression through the use of therapeutic antigene and antisense oligonucleotides [10,11,12,13,14] as well as catalytic nucleic acids [15,16,17,18,19,20,21].

Thereby, synthetic ways to chemically modify nucleic acids analogues have been developed over the past decades in order to obtain a better understanding of the role of DNA in various biological mechanisms or to add unexpected properties to natural processes [22]. For instance, oligonucleotides containing a peptide backbone (peptide nucleic acids, PNA) [23,24,25,26,27], unnatural bases [19,28,29,30,31], or altered phosphate groups [32,33] have been reported.

Similarly, a variety of groups have been attached at the 5′ or 3′ extremities of oligonucleotides, such as fluorophores [34], peptides [35], or metal complexes [36,37,38]. Among the latter, ruthenium complexes have received an increasing interest due to their remarkable redox and photophysical properties, which can easily be modulated by the nature of their ligands. The potential of these metal complexes is widely recognised and they have seen use in electron transfer studies [39,40], catalysis [41], photoprobes [42], or as optical devices [43]. Other chemical properties such as the rate of ligand exchange, the ability of ruthenium to bind biological molecules, the intercalative properties of its ligands and its capacity to interact with DNA in a variety of ways have made ruthenium complexes excellent candidates for cancer treatment and medical diagnosis [44,45,46]. Interestingly, several examples of correlation between different non covalent DNA binding modes of ruthenium complexes and their cytotoxic effects have been reviewed [47]. However, among these numerous applications of ruthenium complexes, only a few studies have been focusing on a covalent bonding interaction with nucleic acids.

In this context, this review will focus only on the elaboration of ruthenium complexes covalently linked to DNA oligonucleotides. We will not discuss the coupling of such complexes with PNA or other DNA analogues [48,49,50,51,52,53,54]. The different synthetic pathways to afford modified oligonucleotides will be discussed and a chronological overview of their most prominent applications in therapy, diagnostics, and as tools for chemical biology will be highlighted. 

## 2. Electron Transfer Enhancement by Ruthenium-Nucleic Acid Complexes

The structural stability of double helical DNA is primarily due π-electron stacking interactions between adjacent base pairs. It is this π-system that enables electron transfer (ET) throughout DNA [55,56,57,58]. Electrons migrate across charge carrying bases in a multistep hopping process, where the transfer rate is dependent on base separation [59]. This primarily occurs through G:C pairs (G-hopping) but when the G-G distance exceeds four bases, A-hopping can be observed (over A:T sites) [60,61,62,63]. Long distance electron transfer is vital in biological processes such as DNA-binding or DNA damage and repair and it can also influence mutations [57,59,64]. Partially due to this, long-range ET through DNA has received considerable interest over the last few decades [64,65,66,67].

**Figure 1:**
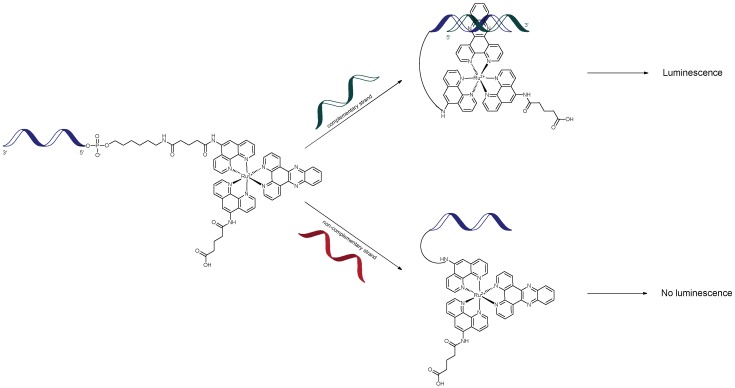
Diagram illustrating the occurrence of intercalation exclusively in the presence of a complementary strand [68,69].

Extensive research on ruthenium-oligonucleotide conjugates has been carried out by the Barton group. It was first shown in 1992 that such complexes could function as sequence-specific molecular light switches for single-stranded DNA when [Ru(phen’)_2_dppz]^2+^ was tethered, via an amide bond, at the 5′-terminus of a 15-mer functionalised with hexylamine. This yielded a Ru-oligonucleotide complex (Ru-ODN) which only showed intense luminescence when in the presence of a complementary strand (Figure 1). This luminescence was positively correlated with intercalation of the complex into dsDNA [68,69]. Enhancement of the luminescence from the Ru-ODN was the result of the lowest excited state of [Ru(phen’)_2_dppz]^2+^, a metal-to-ligand charge transfer (MLCT) transition centred on the intercalating dppz-ligand [70,71,72]. No detectable luminescence was observed from the lone Ru-ODN or upon addition of a non-complementary strand due to the lack of phenazine ring protection from water [68,69]. Such characteristics are important for the sequence-specific targeting of single-stranded DNA. 

Oligonucleotides are well-defined molecular templates which can be further modified with spectroscopically detectable and photochemically active metal-centres at specific locations [73]. These metal-oligonucleotide constructs are useful tools in understanding the mechanism of long-range ET. In this context, Barton et al. investigated in 1993 the long-range electron-donor abilities of a [Ru(phen’)_2_dppz]^2+^-oligonucleotide conjugate. To do so, [Ru(phen’)_2_dppz]^2+^ was tethered at the 5′-terminus of 15-mer functionalised with hexylamine via amide bond formation. Electron-accepting-[Rh(phi)_2_(phen’)]^3+^-oligonucleotides were also prepared [74]. Interestingly, the intense luminescence seen from the ruthenium-bearing 15-mer disappeared when the complementary strand was modified with [Rh(phi)_2_(phen’)]^3+^ (Table 1). Results showed that complete luminescence quenching is only observed when the acceptor is covalently bound within the same duplex as the donor, with the electron-transfer mechanisms occurring over distances as great as 40 Å. The lowest excited state of the rhodium complex, which results from a ligand-to-metal charge-transfer (LMCT), allows this direct photoinduced ET between the two metal centres in the same duplex at a rate of at least 3 × 10^9^ s^−1^ which is comparable to the rate of charge transfer over long distances by the hopping mechanism (10^10^ s^−1^) [75]. Moreover, this is several magnitudes higher than the rates observed in natural DNA duplexes, which typically range from 10^4^–10^6^ s^−1^ [76]. The DNA helix is known to enhance ET processes, specifically ET occurring through π-stacks [77,78,79,80]. To confirm the role that ligand intercalation plays in this quenching, smaller oligonucleotides (5′-CGATTAGC-3′) were metallated with the complexes [Ru(phen)_2_phen’]^2+^ and [Rh(phen)_2_phen’]^3+^ [74]: it is known that the phenanthroline ligand has intercalation depths three times smaller than the dppz ligand [81,82]. Luminescence lifetime experiments yielded similar values independent of the presence of covalent binding between the ruthenium complex and the 8-mer (Table 2). Thus, the lack of intercalation capabilities implied the importance of this non-covalent interaction and the effect of metal-metal distance in the complete quenching of luminescence is demonstrated.

**Figure 2:**
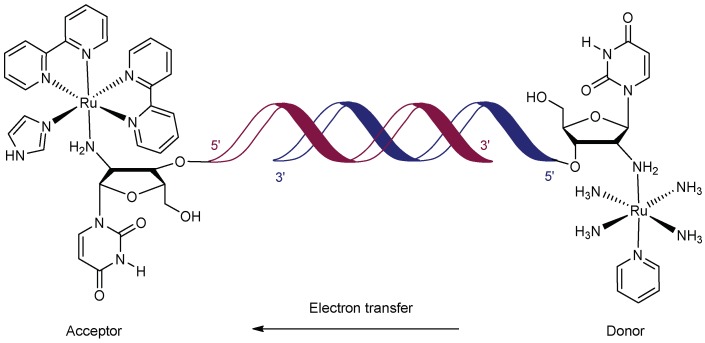
Structure of the duplex DNA (8- or 14-mer) containing ruthenium donor and acceptors [73].

In 1995, Meade and Kayyem used ruthenium complexes as both donors and acceptors for ET through DNA. Two sets of complementary strands (8- and 14-mers) of ruthenium-containing oligonucleotides were synthesised to test the nucleic acid sequence dependency of the electron-transfer process (Figure 2). The acceptor ([Ru(bpy)_2_(im)]^3+^) and donor ([Ru(NH_3_)_4_(py)]^2+^) were covalently attached at the 2’ position of a 5′-terminal ribose following automated solid-phase DNA synthesis. Following this, the two ruthenium-modified oligonucleotides were annealed to yield the duplex DNA with a covalently bonded donor and acceptor. The rate constant for ET between metal centres (Ru-Ru distance = 21 Å) was found to be around 2.5 × 10^6^ s^−1^, which is comparable to that of His39-modified cytochrome c (Fe-Ru distance = 20.3 Å), one of the most efficient protein systems known at this time [73]. 

Further long-range ET studies were completed by the group of Barton in 1997. A flash-quench strategy was employed to demonstrate ET through the DNA double helix [83]. Flash-quenching methods are popular for the study of electron-transfer reactions as they can be used to monitor the oxidation of guanine, the most readily-oxidised base [60,84,85,86]. In this experiment the DNA-bound intercalator [Ru(phen)(bpy’)(Me_2_dppz)]^2+^ was photolysed to enable the ET to an externally bound Ru(III) oxidative quencher, resulting in guanine oxidation in 5′-GG’-3′ sequences. Ru(II)-DNA was prepared by tethering this ruthenium complex to an oligonucleotide duplex containing 5′-GC-3′ or 5-GG-3′, allowing for oxidative damage comparisons [83]. An earlier part of this study showed that positions containing adjacent guanines, such as GG or GGG, are particularly susceptible to oxidation due to the electron-donor abilities of neighbouring guanines. The increased numbers of adjacent guanines was found to promote the oxidation of distant bases [80,83]. In the presence of the externally bound Ru(III), which was generated from Ru(NH_3_)_6_^3+^, selective G-base damage at the 5′-G of a 5′-GG-3′ doublet was observed. In the [Ru(phen)(bpy’)(Me_2_dppz)]^2+^-oligonucleotide containing 5′-GC-3′, there was no preference and all guanine bases experienced equal oxidative damage. For the experiments in the absence of a 5′-GG-3′ guanine doublet, the lack of sequence specificity indicated equal energy sites across all G bases [83], providing further evidence of ET occurring through DNA base pair stacks [80,83]. 

**Figure 3:**
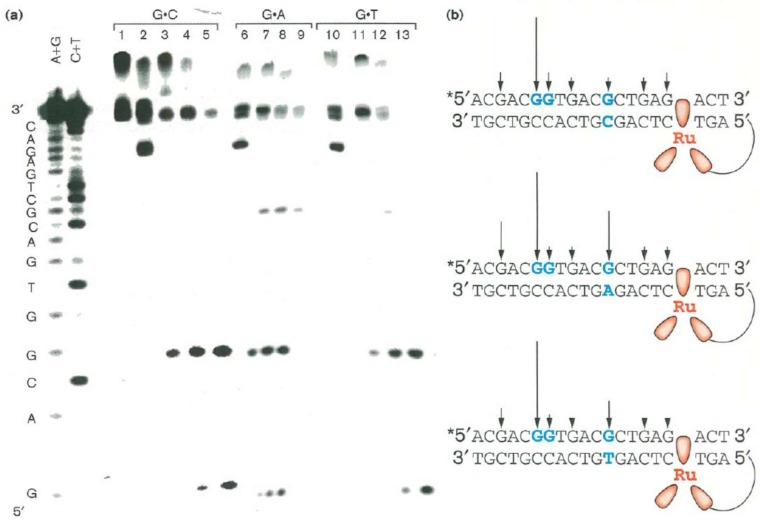
(**a**) PAGE analysis comparing the long-range oxidative damage on Ru(II)-modified DNA in duplexes with a G·A or G·T mismatch with damage in a complementary duplex. (**b**) Sequences of the duplexes used in the analysis. Arrows indicate the position of DNA damage as well as intensity of cleavage. Blue letters indicate the position of the mismatches as well as the GG doublet. Figure taken from [83] with permission.

It was also shown by the Barton group that long-range guanine oxidation was altered by the presence of a single base mismatch. Flash-quench experiments were carried out using [Ru(phen)(bpy’)(Me_2_dppz)]^2+^-DNA duplexes containing G·A or G·T mismatches approximately five base-pairs 3′-downstream from the Ru(II) binding site, as well as a single 5′-GG-3′ doublet around 11 base-pairs away. In the Ru-DNA duplex with a complete base-matched sequence, oxidative guanine damage occurred selectively at the 5′-GG-3′ doublet (lane 3–5 of Figure 3). In the sequence with the G·A mismatch, 16% guanine damage at the 5′-GG-3′ as well as damage of the mispaired guanine was seen (lanes 7–9 of Figure 3). 10% guanine damage at the 5′-GG-3′ site was recorded in the sequence with the more disruptive G·T mismatch, but only minor oxidative damage occurred to the mispaired guanine (lanes 11–13 of Figure 3). Among other things, base mismatches in DNA duplexes lead to localised helix disruption and the structural similarity of the G·A mismatch with the G·C Watson-Crick base pairing can be used to explain the observed difference in guanine oxidative damage between the G·A and G·T mispairings [83]. 

**Figure 4:**
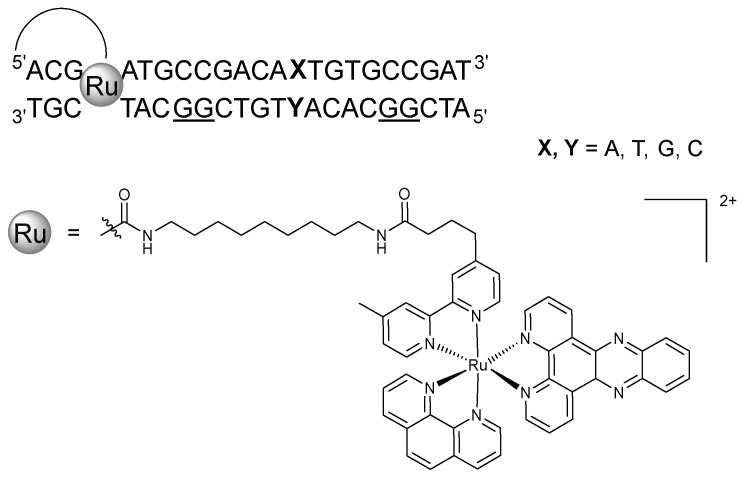
Structure of the Ru(II)-22 mer used in the mismatch experiments. The metal complex is linked, from the bpy’ ligand to the 5′ end of the duplex by a nine-carbon linker. All combinations (16) of base pairs and mismatches were synthesised [87].

In 2001, Bhattacharya and Barton showcased the possibility of controlling long-range charge transport by manipulation of base-pairs dynamics. [Ru(bpy’)(dppz)(phen)]^2+^ was covalently bonded to the 5′-terminus of a series of 22-mers by a nine-carbon linker. Each sequence contained two 5′-GG-3′ sites in the complementary strand, between which a single base mismatch was introduced (Figure 4). All mispair-containing sequences were synthesised and the efficiency of electron transport through the base mismatches was determined by comparing the guanine oxidation of the 5′-G at the two 5′-GG-3′ sites: the ratio of distal/proximal damage. The value was found to be different for all base-pairings with the general trend of G·C ≈ G·G ≈ G·T ≈ G·A > A·A > C·C ≈ T·T ≈ C·A ≈ C·T, with the purine-purine mispairs yielding the highest ratios. The distal:proximal guanine oxidation trend was compared with various duplex properties, but the damage ratios correlated most closely with base-pair lifetimes. The lifetimes of the Ru-ODNs containing G·G, T·T, A·A, and C·C pairings were determined by monitoring the imino proton exchange by ^1^H NMR and comparing with that of the G·C Watson-Crick base pair (τ_ex_(G5NH) = 18 ms). The Ru-DNA duplex containing the G·G mismatch had the longest lifetime followed by the A·A, C·C, and T·T. The lifetime of the T·T mispair was four times shorter than that of G·G, yielding the lowest distal:proximal oxidation damage [87].

**Scheme 1:**
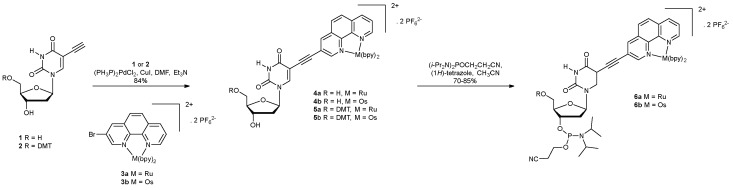
Synthesis of phosphoramidites **6a** and **6b [88]**.

Hurley and Tor first reported the site-specific incorporation of metal complexes during the solid-phase synthesis oligonucleotides in 1998. This overcame restrictions associated with terminal-functionalization of oligonucleotides and removed the need of exposure to reactive metal precursors often associated with other approaches. In their approach, high-yield solid-phase phosphoramidite chemistry was utilised: the phosphoramidites were prepared by phosphitylation of their corresponding ruthenium and osmium containing protected nucleosides (Scheme 1). These modified 2’-deoxyuridine building blocks could then be used in automated solid-phase DNA synthesis [88].

**Figure 5:**
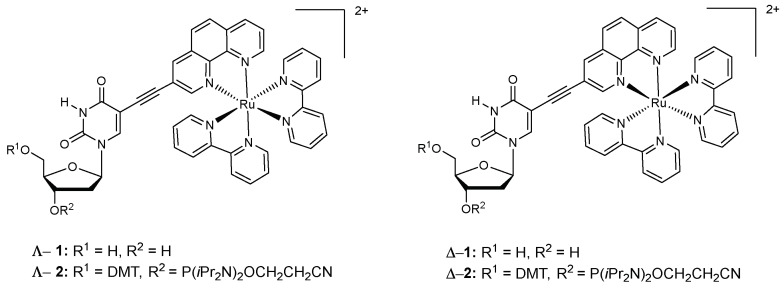
Chemical structures of the nucleosides containing the diastereomer Λ-**1** and Δ-**1** and their corresponding phosphoramidites (Λ-**2** and Δ-**2**).

A similar methodology was also applied to synthesise diasteromerically pure Ru(II)-ODNs. The absolute configuration of the Ru-oligonucleotide conjugates was controlled by using enantiomerically pure nucleoside phosphoramidites. [Ru(bpy)_2_(3-bromo-1,10-phenanthroline)]^2+^ was covalently attached to the 5-position of the nucleobase of 2’-deoxyuridine by a Sonogashira cross-coupling reaction, followed by the synthesis of the Ru(II)-nucleosides (Δ-**1** and Λ-**1**) and the respective phosphoramidites (Δ-**2** and Λ-**2**) (Figure 5) [89,90,91]. The CD spectra of the nucleosides showed that both configurations emitted at the same wavelength (632 nm) upon MLCT-band excitation and the lifetime of these excited-states was also the same (1.07 μs). It is not until incorporation into the respective oligonucleotides that the diastereomeric effects are visible. Specifically, the CD spectrum of Λ-Ru(II)-ODN is dominated by a large band at 300 nm which is the result of the polypyridine ligand and heterocyclic base transitions overlapping. There was also a minimal difference in thermal stability between the two isomers [91].

In a further study, the donor/acceptor interactions between the same ethynyl linked Ru(II)-nucleoside and its osmium analogue were investigated. [Ru(bpy)_2_(3-ethynyl-1,10-phenanthroline)]^2+^ was fixed at the 5′ terminus of a 19-mer and the position of the acceptor, [Os(bpy)_2_(3-ethynyl-1,10-phenanthroline)]^2+^, on the complementary strand was incrementally distanced by three base pairs, from 3 (16 Å) to 18 (60 Å) base pairs apart (Table 3 and Table 4). This was achieved using the corresponding epimeric phosphoramidites for solid-phase DNA synthesis. In quenching studies similar to those previously described in this review, a positive correlation between Os(II)-Ru(II) distance and average Ru(II) excited-state lifetime was seen. 

**Figure 6:**
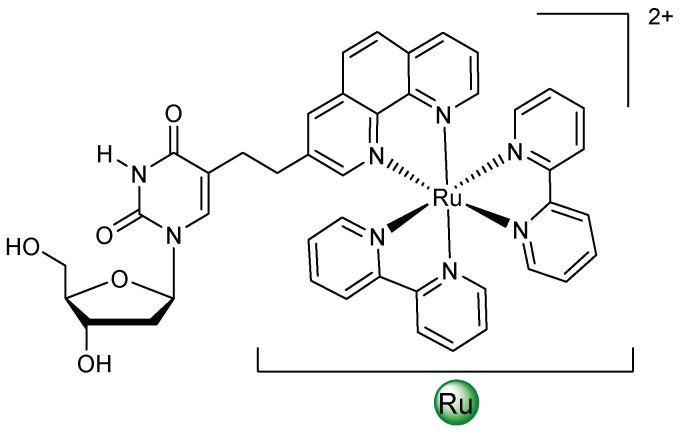
Structure of the Ru-containing-Uridine.

The principal aim of this study was to determine the primary mechanism for the ET between these donor/acceptor metal centres. To do so, the ethynyl linked Ru(II)-donor was replaced with a saturated two-carbon linked complex to allow for increased flexibility (Figure 6). The same trend of distance dependent excited state lifetime for the Ru(II) donor was found to be true as for the more rigid nucleoside analogues. The energy transfer between the donor and acceptor was monitored using both steady-state and time-resolved techniques. This data was analysed in coherence with the Förster dipole-dipole and the Dexter electron exchange mechanisms. A significant discrepancy was observed between experimentally determined quenching data and theoretical Förster curves when the more rigid Λ-**2** and Δ-**2** building blocks were used. The rigidity of the linker arms was believed to affect the geometric factor *κ*^2^ used in the calculation of the critical Förster radius *R*_o_ in an unpredictable manner. When the more flexible linker was used, *κ*^2^ was closer to the typical value ascribed to this parameter and the correlation between calculated and experimental Förster curves was much higher. Therefore, the primary mechanism of the energy transfer was determined to be mainly based on Förster dipole-dipole interactions since efficiency decreased with increasing donor/acceptor distance. However, a minor contribution of the Dexter electron exchange mechanism, which operates at shorter distances, could not be entirely ruled out due to the variation from idealised Förster behaviour [92].

**Scheme 2:**
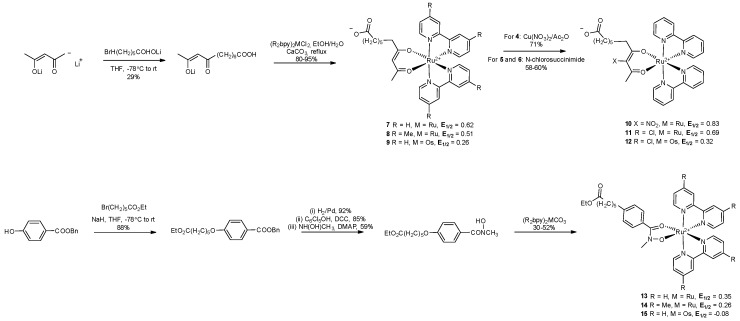
Synthesis and electrochemical data of acetyacetonate complexes (**7**–**12**) and hydroxamate complexes (**13**–**15**) [95].

**Scheme 3:**
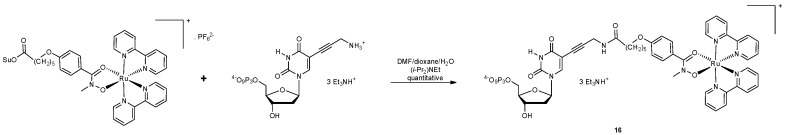
Synthesis of triphosphate **16** (dRuTP) [95].

**Figure 7:**
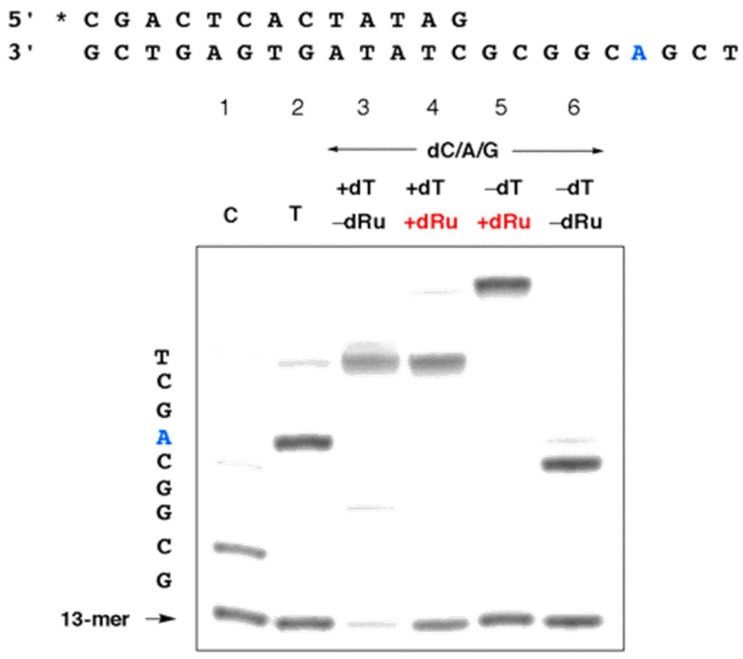
Enzymatic incorporation of dRuTP. Primer extension experiments using the Klenow fragment of DNA polymerase I resolved on a 20% PAGE. Lanes 1 and 2: control reactions missing one natural triphosphate (dCTP or TTP); lane 3: control reaction with all four natural dNTPs; lane 4: four natural dNTPs and dRuTP; lane 5: dRuTP used instead of TTP; lane 6: control reactions without TTP and dRuTP. Figure taken from [95] with permission.

Modified nucleoside triphosphates can be used to incorporate functional groups and particularly metal complexes into DNA enzymatically (vide infra) [93,94]. This approach was used by Weizman and Tor in 2002 to develop metal-containing redox tags. Complexes of the general formula [Ru(bpyR_2_)L_2_]^2+^ were used due to the kinetic inertness, chemical stability, and ligand-based tunability of electrochemical potentials (E_1/2_) associated with ruthenium(II) polypyridyl complexes. There were three building blocks used in the synthesis: a substituted 2,4-pentadione or hydroxamic acid with a hydroxamate functionalised linker, a bis-substituted Ru^2+^ precursor and a modified nucleotide (Scheme 2 and Scheme 3). The Ru(II) complexes were bound by their succinimide ester to 5-propargylamino-dUTP (5-(3”-aminopropynyl)-2’-deoxyuridine-5′-triphosphate) via an amide bond (Scheme 2 and Scheme 3). Analysis of the primer extension reactions with the resulting modified triphosphate dRuTP into DNA clearly demonstrated that the modified triphosphate acted as a good substrate for the Klenow fragment of DNA polymerase I and that the Ru(II)-DNA had the expected lower electrophoretic mobility compared to unmodified strands (Figure 7) [95]. 

## 3. Nanomaterials Based on Ruthenium-Oligonucleotide Conjugates

**Scheme 4:**
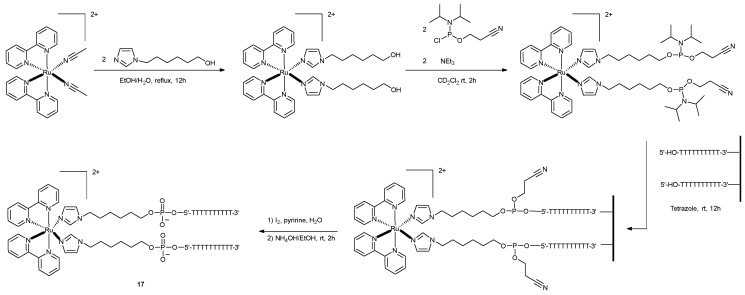
Synthetic pathway for the Ru-DNA conjugate **17**.

The predictable behaviour of DNA has made it a useful scaffold to organize molecules into high-order 2- and 3-dimensional structures such as stars, disks, cubes, or even boxes [96,97]. In order to further expand the structural diversity accessible to nucleic acids, Sleiman et al. reported in 2001 the first synthesis of a Ru(II) complex linked to a branched oligonucleotide (**17**, Scheme 4), in which two DNA strands are covalently linked to the complex [98]. A *cis*-[Ru(bpy)_2_(imidazole)_2_]^2+^ complex is linked through hexyl spacers to two parallel dT_10_ oligonucleotides. The preparation of this molecule was made by a convergent solid phase strategy [99] described in Scheme 4. First, a stable ruthenium bis-phosphoramidite branching complex was synthesised which then could be appended on a dT_10_ oligonucleotide obtained by solid-phase synthesis. Due to the presence of two phosphoramidite moieties on the ruthenium complex, the 5′-termini of oligonucleotides needed to be in close proximity to each other on the solid support and therefore a high-density controlled pore glass (CPG) support was used.

The Ru-DNA conjugate **17**, as a branched oligonucleotide, has the ability to specifically hybridize complementary single stranded DNA or RNA but could also be associated with analogues of molecule **17** containing mixed DNA sequences to create new DNA nanostructures [100]. Moreover, the presence of a ruthenium complex confers redox and luminescent properties to this DNA structure. In this context, the authors determined the hybridization efficiency of **17** with its complementary DNA dA_10_ by thermal denaturation experiments. When one equivalent of dA_10_ was added, the formation of a duplex with one of the complementary strands of the branched oligonucleotide was observed with a melting temperature (*T*_m_ value) of 21 °C, which is close to that of a natural Watson-Crick dA_10_/dT_10_ duplex (*T*_m_ = 22 °C). When two equivalents of dA_10_ were added, both strands of **17** were successfully hybridised (*T*_m_ = 26 °C). Despite these favourable assets, the presence of the two monodentate imidazole ligands along with the flexible hexyls spacers prevented control of the self-assembly process since no nanostructure containing two ruthenium complexes could be obtained. 

**Figure 8:**
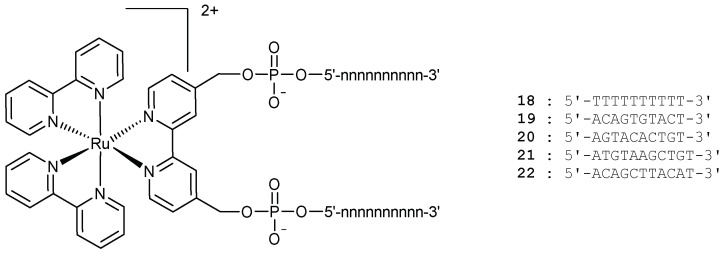
Structures of the Ru complex linked branched oligonucleotides **18**–**22**.

In 2004, the same group applied their strategy to the elaboration of more rigid ruthenium branched oligonucleotides **18**–**22** (Figure 8) [101].

**Scheme 5:**
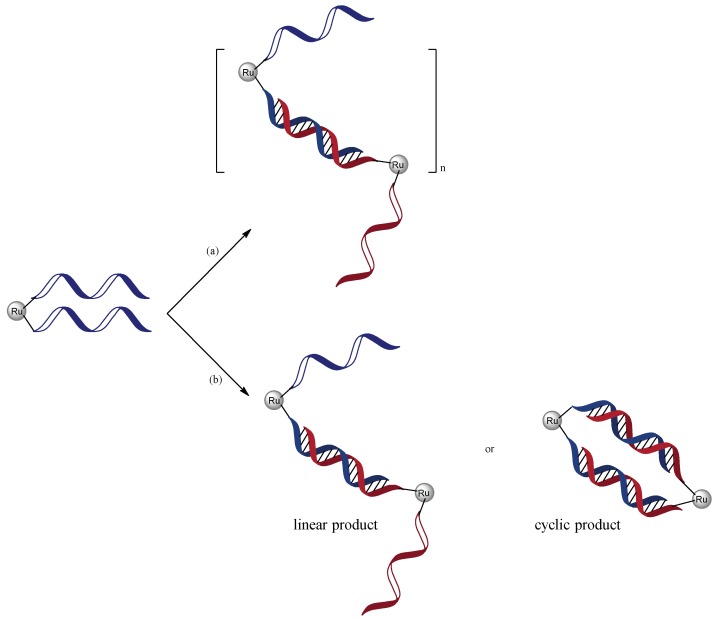
Hybridization of **21** (blue) by the addition of its complementary Ru-DNA strand **22** (red). (**a**) 90 °C to 4 °C, overnight; (**b**) 4 °C, overnight [101].

These molecules were able to form stable duplexes upon hybridization with their complementary strands, with a slight increase in their melting temperatures compared to the unmodified Watson-Crick duplexes (Δ*T*_m_ of 1 to 4 °C). In terms of self-assembly properties, when an equimolar solution of **21** with its complementary ruthenium-containing oligonucleotide **22** was heated up to 90 °C, and then cooled down to 4 °C, a polymeric Ru-DNA species was detected. Under milder conditions (4 °C, 12 h), a dimer was created, which could be linear or cyclic (Scheme 5). 

In order to characterize the nature of the dimer, an enzymatic digestion experiment was conducted. Mung Bean Nuclease has the ability to degrade selectively oligomeric species that contain single DNA strands. The dimer was found to remain unmodified upon this experiment, showing that the self-assembly of two ruthenium branched oligonucleotides leads to a cyclic nanostructure, addressable by means of light or electrical energy. 

**Figure 9:**
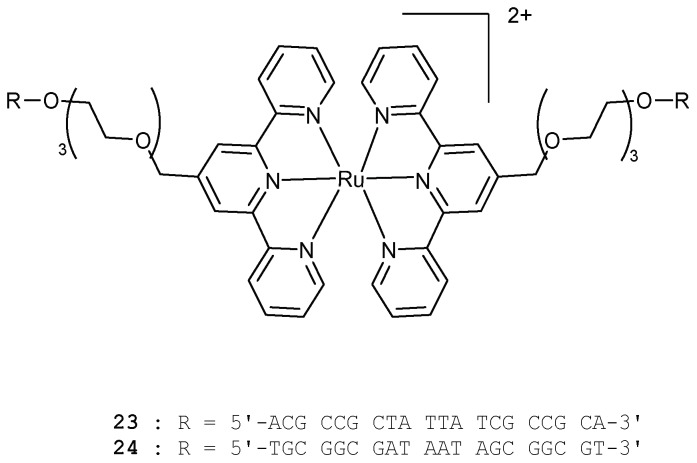
Structures of the Ru complex linked branched oligonucleotides **23**, **24**.

At the same period, Stewart and McLaughlin developed ruthenium bis(terpyridine) complexes linked to two identical 20-mer DNA sequences through triethylene glycol linkers (**23**, **24**, Figure 9) [102].

A di(ethylene glycol) linker was added to a terpy ligand and the corresponding Ru(II) complex was synthesised [103]. The corresponding phosphoramidite molecules were then prepared and **23**, **24** were synthesised through DNA automated synthesis. Due to the geometrical arrangement of these complexes, they are expected to form linear arrays under hybridisation. The complementary DNA sequences **23** and **24** were chosen so as to create a linear array in which ruthenium complexes are placed at a fixed and regular distance. With a 2:1 ratio or greater of **23**:**24** (or **24**:**23**), a trimer **23**=**24**=**23** (or **24**=**23**=**24**) was found as a major product. By reducing the number of equivalents, the length of the linear arrays was found to increase and products were analysed by non-denaturing PAGE experiments. The longest array was obtained for a 1:1 reaction and the procedure developed could be used to place ruthenium complexes at regular distances in linear arrays. 

**Figure 10:**
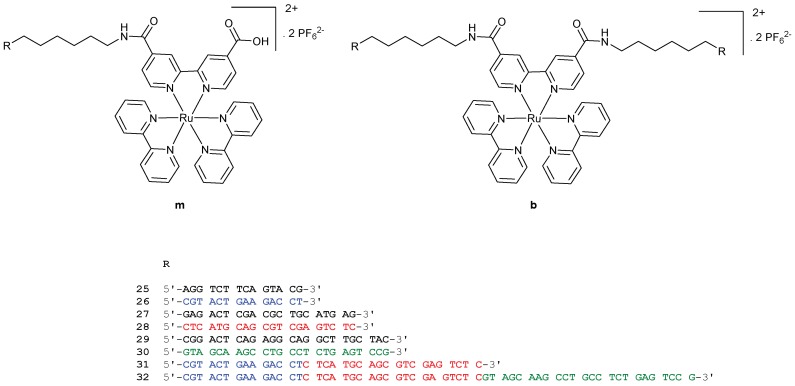
Structures of **m25**–**m32** mono Ru-DNA and **b25**–**b32** bis Ru-DNA.

A few years later, Sartor et al. developed a new methodology to construct linear nanoassemblies of ruthenium-DNA conjugates in a programmable manner for applications such as symmetrical nanowires [104]. First, the synthesis of ruthenium-DNA conjugates is based on the coupling reaction of a Ru(II)(2,2’-bpy)_2_(4,4’-dicarboxy-2,2’-bpy) complex synthesised as described in [105] with commercially available oligonucleotides bearing an amino hexyl linker at the 5′ position. As shown on Figure 10, 8 mono Ru-DNA (**m25**–**m32**) and 8 bis Ru-DNA (**b25**–**b32**) molecules were prepared by this methodology. Sequences **25**/**26**, **27**/**28**, and **29**/**30** are complementary to each other. 

**Figure 11:**
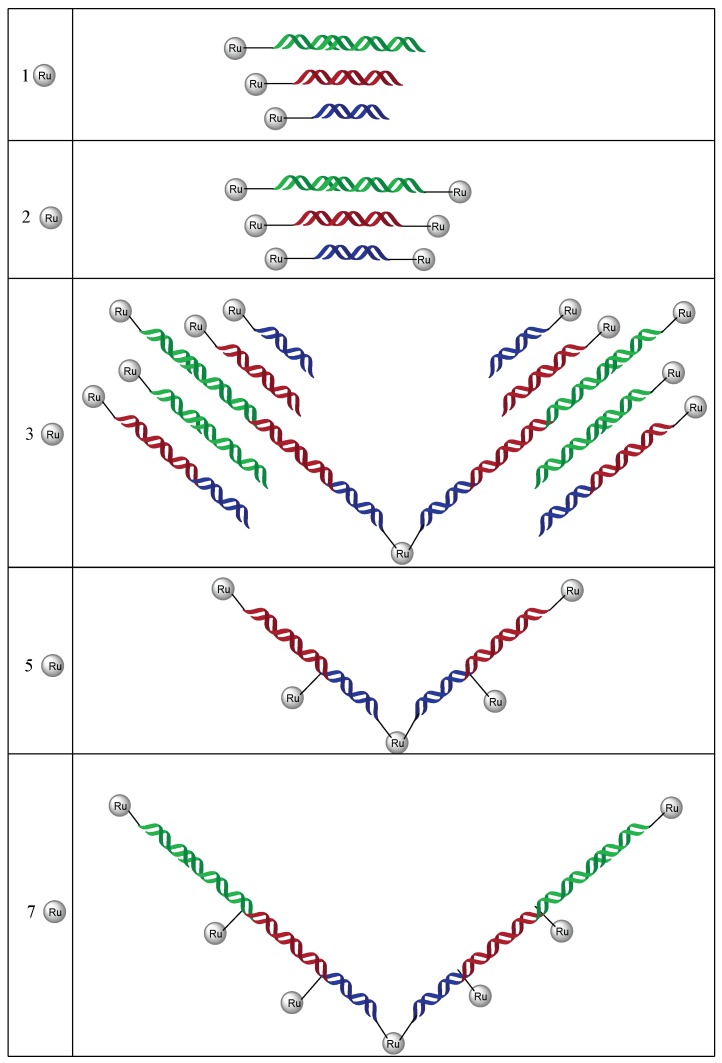
Structures of the linear DNA nanoassemblies with one to seven ruthenium complexes [104].

With these Ru-DNA building blocks, a variety of linear assemblies could be created by preparing a solution of mono or bis Ru-DNA conjugates with their respective complementary complexes. The hybridization process promoted assemblies with one to seven ruthenium complexes (Figure 11). The distance between two ruthenium complexes could easily be modulated due to the different sizes of the oligonucleotide sequences. Interestingly, hybridisation of Ru-DNA **31b** and **32b** could be achieved with the use of single complementary DNA strands followed by a complementary mono-Ru-DNA to give a nanoassembly with three ruthenium complexes. This could also be achieved with only mono or bis Ru-DNA to increase the incorporation of ruthenium complexes. **31b** was then hybridised with two equivalents of **25m** and **27m** (incorporation of 5 Ru) and **32b** with two equivalents of **25m**, **27m**, and **29m** (incorporation of 7 Ru).

This way of synthesis allows the programmable assembly of complementary Ru-DNA molecules in a linear manner. The number and position of ruthenium complexes incorporated in the DNA backbone can be easily controlled. 

**Figure 12:**
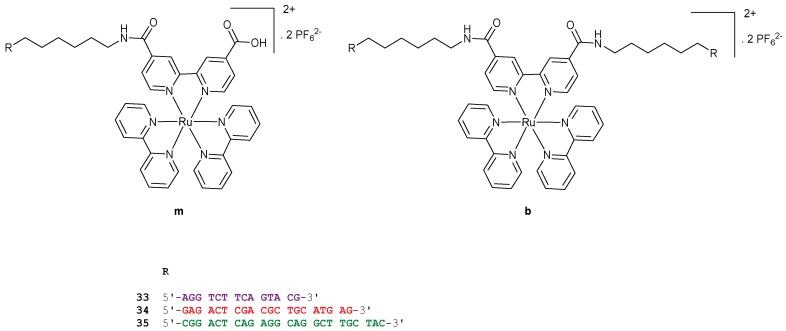
Structures of **m33**–**m35** mono Ru-DNA and **b33**–**b35** bis Ru-DNA.

This work paved the way to the elaboration of other patterns. For instance, in 2013, DNA three-way junction-ruthenium complexes assemblies were created [106]. As a proof of concept, a ruthenium free three-branched DNA junction was first formed by hybridization of three complementary oligonucleotide sequences (**1**, **2**, and **3**) in which a dodecyl linker is present (Figure 12).

Oligonucleotides **1**–**8** were then used to incorporate different numbers of mono-Ru-DNA (**m33**–**m35**, Figure 12) at the periphery of the three-way junction (Figure 14). The introduction of ruthenium complexes at every end was believed to lock the constructs into three-point star motifs. 

The authors successfully controlled the number of ruthenium complexes that could be incorporated at the periphery and were able to modulate the length of the binding arms. 

**Figure 13:**
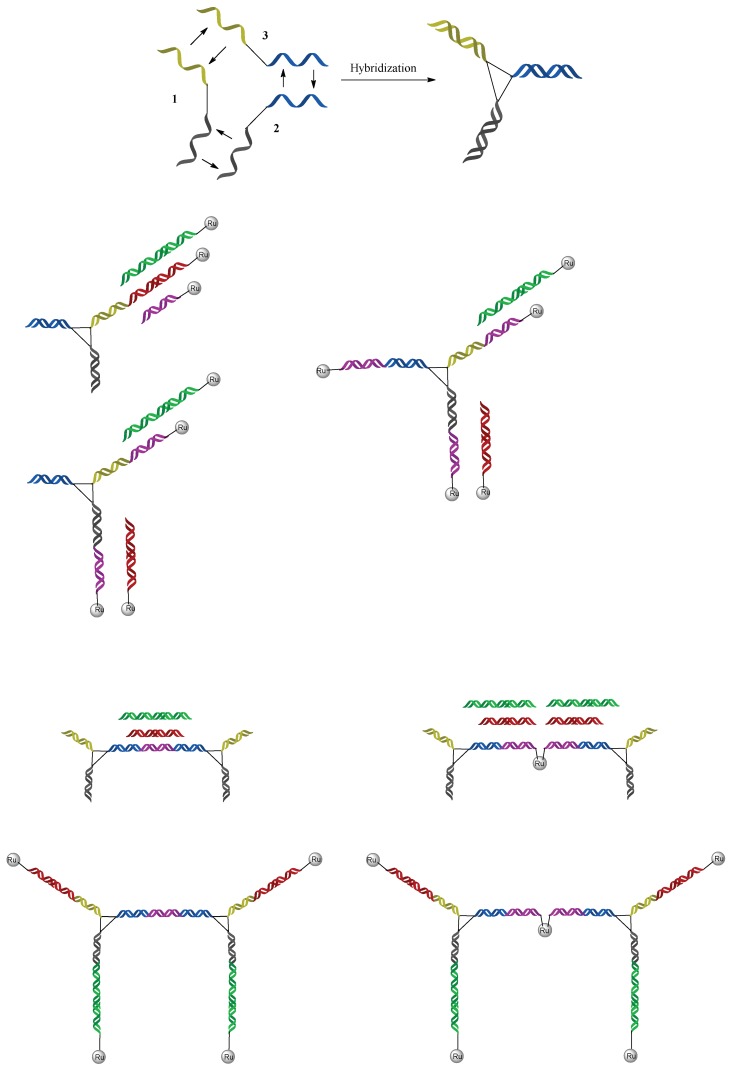
Synthesis of the three-way junction by hybridization of DNA sequences **1**, **2**, and **3** (black lines represent the dodecyl linkers) and structures of three-way DNA junctions with mono Ru-DNA and double-star assemblies [106].

The use of a bis-Ru-DNA construct permitted in a second step to connect two three-way junctions and to elaborate more complex structures (Figure 13). With a bis-Ru-DNA at the centre and no mono-Ru-DNA at the periphery of the three-arm star-shaped junctions, the efficiency formation of the assemblies decreased. On the other hand, other mono-Ru-DNA could be incorporated successfully at the periphery of the three-arm star-shaped junctions. However, when the structures were visualised by polyacrylamide gel, the size limit for the PAGE seemed to be reached suggesting the formation of rather large constructs. These studies demonstrate that the incorporation of ruthenium complexes at the periphery of a three-way junction can be used to study the thermal stability and the global structure of such assemblies. 

## 4. Ru-DNA and the Antigene and Antisense Strategies

The use of the complementary properties of oligonucleotides has enabled the construction of complex assemblies at the nanoscale level. Yet, the specific binding of nucleic acid analogues can also be used to target DNA through the formation of a triple helix. In the antigene strategy, a triplex-forming oligonucleotide (TFO) blocks the transcription of DNA into mRNA and, therefore, acts directly on the regulation of gene expression, potentially knocking down the expression of disease-associated proteins [107,108].

**Scheme 6:**
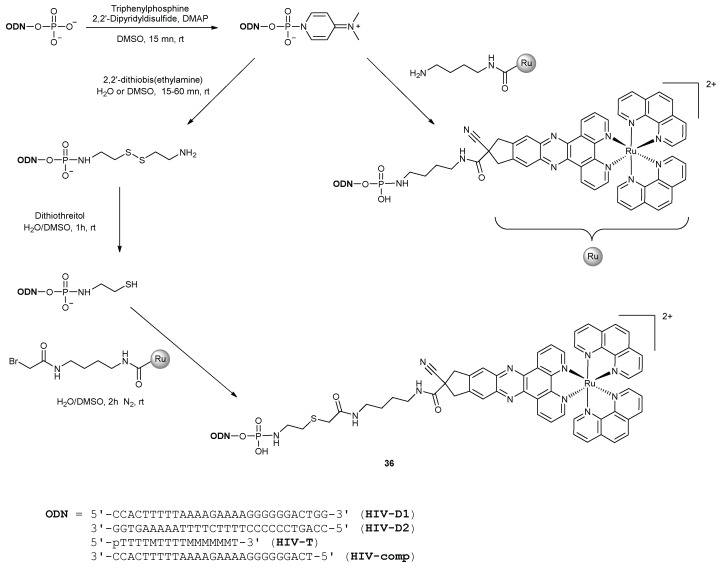
Preparation of oligonucleotide-Ru conjugates. 5′p = 5′-phosphate group, M = 5-methylcytosine [109].

In this context, Hélène et al. took an interest in the Δ and Λ enantiomers of the [Ru(phen)_2_dppz]^2+^ complex [109]. These molecules were previously reported as good double-stranded DNA binding agents, due to the intercalation of the ligands between DNA base pairs [71,72,110,111]. These interesting properties could be used for the stabilisation of DNA triple helices by conjugation of a Ru complex to the 5′-end of a TFO [112,113]. The presence of the ruthenium would also add photophysical properties, such as luminescence, long-distance electron transfer, or photocleavage to the resulting triple helices. Such Ru-oligonucleotides could be used in the context of the antigene therapy, but also as photosensitizers for directed photodamage of DNA, or for the study of DNA binding. Thus, two efficient synthetic approaches were developed to link Δ and Λ-[Ru(phen)_2_dppz]^2+^ at the 5′-end of oligonucleotides (Scheme 6) [114]. In the first approach, an alkylated ruthenium complex derivative is coupled to a thiol-modified oligonucleotide. The second synthetic route consists in the direct linkage of an amino-containing ruthenium complex with an oligonucleotide bearing an activated 5′-phosphate group. Even if the linkage of **ODN** to Ru(II) complex through an amine bond was effective, the thiol alkylation provided a better yield and the authors used **36** for further experiments. 

The luminescence of the Δ and Λ enantiomers of the ruthenium complex linked to **HIV-T** oligonucleotide was investigated. Neither of the two moieties was luminescent in aqueous solution. Upon formation of the triple helix by addition of double-stranded DNA (composed of **HIV-D1** and **HIV-D2**), the luminescence of the Δ enantiomer was increased by 6–10 times, unlike the Λ enantiomer, which showed no enhancement of luminescence. This phenomenon was previously reported for double-stranded DNA and can be explained by a difference in the geometry of intercalation. The geometry of the Λ enantiomer is more favourable to nonspecific inter- or intramolecular stacking interactions between the dppz ligand and the oligonucleotide bases [72,111,115]. 

The stability of triple-stranded DNA formed with **HIV-D1**, **HIV-D2**, and Λ**-HIV-T-Ru** was then investigated. Thermal denaturation experiments showed the stabilisation of the triple helix with the attachment of [Ru(phen)_2_dppz]^2+^, with a stabilisation reaching Δ*T*_m_ = 12 °C. Unmodified **HIV-T** oligonucleotide was found to form less stable triplexes than **HIV-T-Ru**. Hence, competition studies revealed that the latter was able to displace the unmodified oligonucleotide from triplex structures. This study showed the effective intercalation of a Ru(II) complex between DNA base pairs, leading to a strong stabilisation of the triple helix, with an increase of its lifetime. The intense luminescence of the ruthenium complex upon formation of the triplex could be further used to study the kinetics of DNA binding or as a probe to study the hydrophobicity of an environment.

**Scheme 7:**
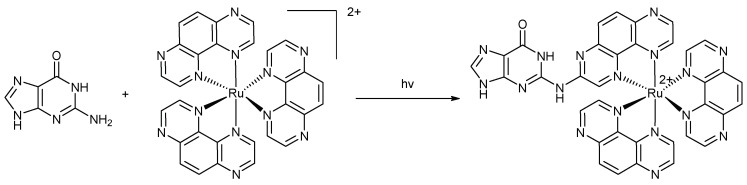
Formation of a covalent photoadduct between guanine and [Ru(TAP)_3_] under irradiation [125,126].

Another promising class of therapeutic agents are nucleic acids analogues binding directly to the encoding mRNA of a defined, disease-associated protein. Indeed, in the antisense strategy a chemically modified oligonucleotide (antisense oligonucleotide, ASO) recognises a stretch of mRNA through complementary base-pairing and the resulting steric blocks prevents translation [116]. Alternatively, ASOs can recruit RNase H which catalyses the cleavage of the RNA part of the ASO-mRNA duplex [117,118]. The development of molecules suited for the antisense strategy and gene silencing could serve as new anticancer agents [119]. Thereby, we will focus on complexes bearing a TAP ligand as non-intercalative agents. The excited states of these complexes are capable of oxidising free guanine or guanine nucleotides in DNA oligonucleotides [120] by a charge transfer process followed by a back electron transfer [121,122,123]. By measuring the reduction potential of several complexes, it was shown that at least two oxidising π–deficient TAP ligands were required in a complex to react with guanine [124]. In order to determine the nature of the reaction between guanine and these ruthenium complexes, the study of [Ru(TAP)_3_]^2+^ with guanosine monophosphate was carried out. This showed the formation of a covalent photoadduct between the exocyclic amine of the guanine nucleobase and one of the TAP ligands (Scheme 7) [125,126]. The mechanism involves the radical recombination of the protonated reduced ruthenium complex [127,128] and the deprotonated radical cation of guanine and rearomatisation of the nucleobase leads to the formation of the observed photoadduct. Interestingly, no bonding to the O6 centre of guanine was observed despite that radicals centred on O6 are usually the most stable.

In 2009, Kirsch-De Mesmaeker et al. applied this knowledge to gene silencing applications by synthesising ruthenium complexes bearing two TAP ligands [129]. Under light irradiation, and in the presence of an oligonucleotide duplex containing a guanine base on each strand, a photo-cross-linking reaction occurred. As a result, the exonuclease activity was inhibited and the DNA damaged [130]. 

**Figure 14:**
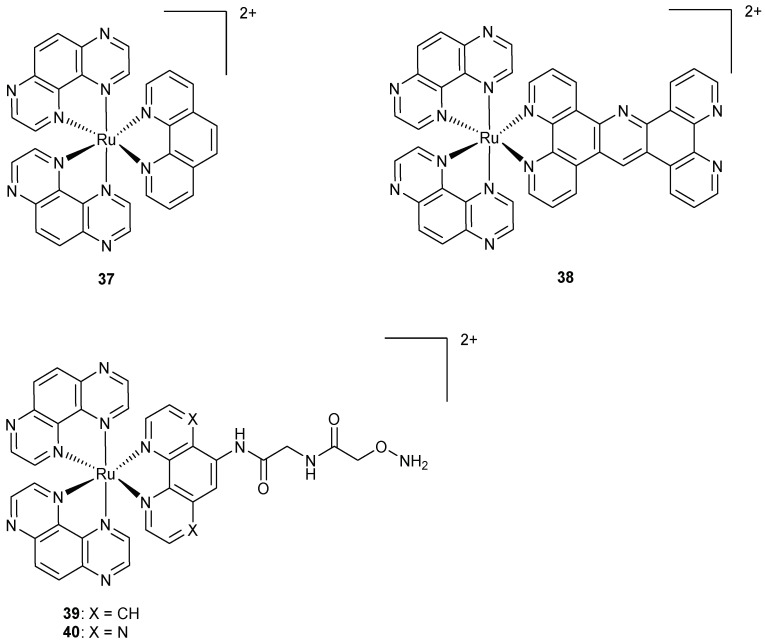
Structures of the Ru complexes [Ru(TAP)_2_Phen]^2+^ **37,** [Ru(TAP)_2_(TPAC)]^2+^ **38** [131], [Ru(TAP)_2_phen’]^2+^ **39**, and [Ru(TAP)_2_TAP’]^2+^ **40** [132].

Preliminary experiments showed that **37** and **38** (Figure 14) exhibited a moderate affinity for DNA (K = 3.9 × 10^4^ M^−1^ and K = 1.5 × 10^6^ M^−1^, respectively) [129]. Molecular modelling simulations showed an incomplete intercalation of the TPAC ligand into the base pairs of duplex DNA, revealing that **38** would be more constrained within the duplex. PAGE analysis of different duplexes containing a ^32^P-labeled strand in the presence of the ruthenium complexes and under irradiation was then carried out in order to study the photo-cross-linking process. Two photoadducts were found for complex **37**: one corresponding to a photo-cross-linking reaction between the complex and the two strands of the duplex and the second within a single strand. For complex **38**, no photo-cross-linking reaction could be detected under the same conditions. This phenomenon was ascribed to the intercalation of **38** into the stacked DNA base pairs which prevents the mobility of the complex inside the helix. The study of **38** showed that intercalative ligands do not possess the most favourable geometry to induce a photo-cross-linking reaction. 

As **37** showed promising results for photo-cross-linking applications, Kirsch-De Mesmaeker and her group linked **37** to an oligonucleotide through an oxime bond. The oxyamino-aldehyde coupling reaction applied to oligonucleotides was first described by Lhomme and co-workers in 2000 to link an aldehyde containing oligonucleotide to a fluorophore bearing an oxyamine function, to give a stable conjugated oligonucleotide [133]. The oxime bond strategy was found to be a rapid, selective, and regiospecific manner to introduce functionalities at a preselected position inside the sequence of an oligonucleotide. This technique was then extended to the ligation of oligonucleotides with peptides, carbohydrates, glycopeptides, and PNA [134,135,136,137].

**Scheme 8:**
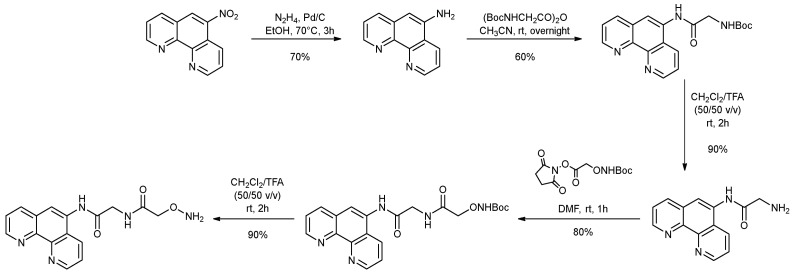
Preparation of the oxyamino phenanthroline derivative [138].

In 2003, Defrancq and co-workers applied this strategy to covalently couple an amino-modified phenanthroline derivative at the 5′ or the 3′-end of an oligonucleotide [138]. The oxyamino phenanthroline derivative synthetic route is presented in Scheme 8.

The oligonucleotide bearing an aldehyde at the 5′ or 3′-end was synthesised using standard automated solid-phase DNA synthesis. The conjugation of the two moieties was then carried out at pH 4.5 in ammonium acetate buffer, as slightly acidic conditions are needed to synthesise the oxime bond with efficiency through an addition-elimination mechanism. As two aldehyde-containing oligonucleotides can react with one aminooxy group [139], two equivalents of the phenanthroline derivative were used to minimise the formation of this by-product. This successful linkage of a phenanthroline derivative paved the way to the coupling of ruthenium complexes to oligonucleotides. In 2007, Defrancq et al. reported the first synthesis of Ru(II)-oligonucleotide complexes through the formation of an oxime bond [132]. [Ru(TAP)_2_phen’]^2+^ (**39**, Figure 14) and [Ru(TAP)_2_TAP’]^2+^ (**40**, Figure 14), where TAP’ or phen’ are the corresponding ligands bearing an aminooxy function were synthesised. 

**Figure 15:**
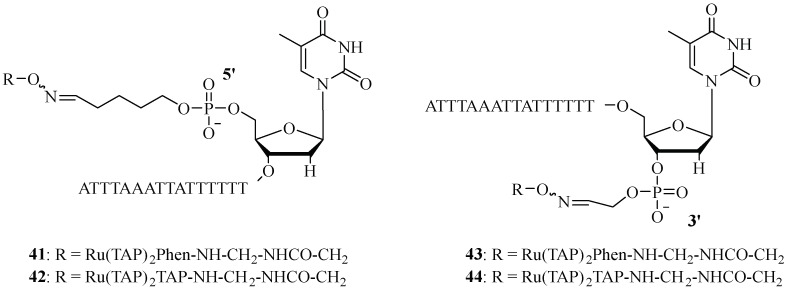
Structures of the oligonucleotide-Ru complexes **41**–**44** [132].

First, the phen and TAP ligands with Boc-protected aminooxy function were prepared by application of the synthetic pathway described above. The ligands were then reacted with [Ru(TAP)_2_(H_2_O)_2_]^2+^ [140] to give the corresponding complexes in 70% overall yield. The Boc function was then deprotected with a 1N HCl solution to avoid the use of TFA which, as an ambident reagent, could substitute a TAP ligand. Once the highly reactive aminooxy group was deprotected, 2 equivalents of the complexes were reacted with the aldehyde-containing oligonucleotides under acidic conditions. The expected products **41**–**44** (Figure 15) were obtained with an isolated yield of 40% for both a coupling at the 5′- or 3′-terminus of the oligonucleotide. Worthy of note, these conditions are compatible with the stability of the ruthenium complexes, as no degradation occurred during the reaction or the purification. 

**Figure 16:**
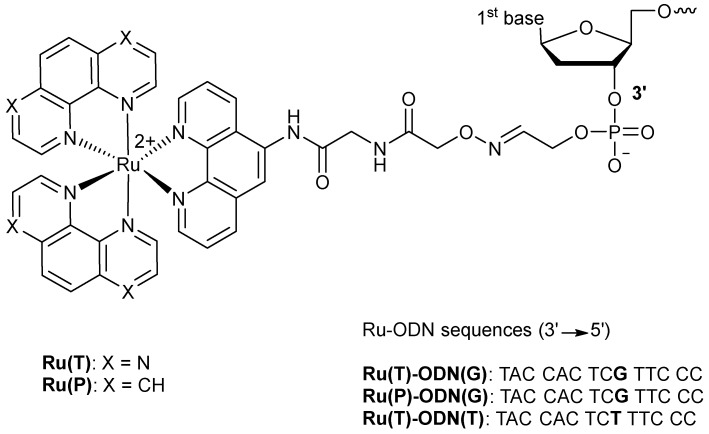
Structures of the Ru-ODN molecules [141].

Following this methodology, Kirsch-De Mesmaeker and her group designed two polyazaaromatic ruthenium complexes to study the impact of a guanine base inside a ruthenium-oligonucleotide strand on photo-cross-linking reactions. A photoactive [Ru(TAP)_2_phen”]^2+^ **Ru(T)** and a non-photoactive [Ru(phen)_2_phen”]^2+^ **Ru(P)** were synthesised [141]. The phen” ligand corresponds to *N*-(2-(1,10-phenanthrolin-5-ylamino)-2-oxoethyl)-2-(aminooxy)acetamide and allows the linkage between the complexes and the 3′-end of modified oligonucleotides through an oxime bond. The attachment of the complexes at the 5′-end of ODNs was not considered as studies have shown that the efficiency of the photo-cross-linking process was higher on the 3′-end due to the suppression of the photodechelation process [142]. Three Ru-ODN sequences were created with two types of ODNs : (1) with a guanine base **ODN(G)**; or (2) without a guanine base **ODN(T)** (Figure 16). **ODN(T)** was used as a negative control.

**Figure 17:**
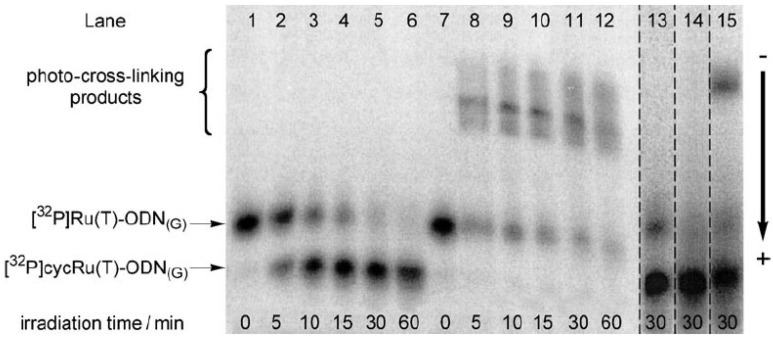
PAGE analysis of the photochemical behaviour of **Ru(T)**-**ODN(G)** in the absence (lanes 1–6) and in the presence (lanes 7–12) of the complementary target strand. Lanes 13, 14, and 15 correspond to **Ru(T)**-**ODN(G)** that was irradiated for 30 min in the presence of non-complementary sequences. Figure taken from [141] with permission.

**Scheme 9:**
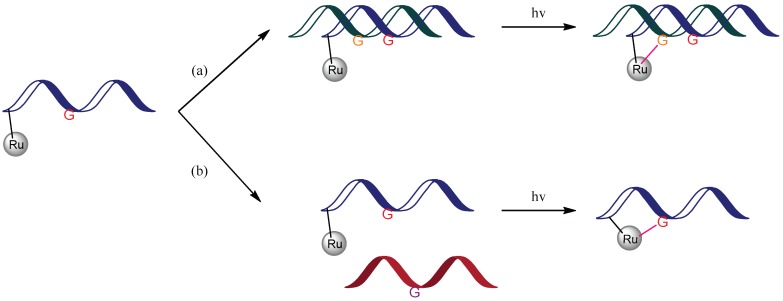
Mechanism of action of **Ru(T)-ODN(G)** molecules. (**a**) With a complementary target, interstrand photoreaction. (**b**) Without a target, or with a non-complementary strand, intramolecular photoreaction [141].

A similar intramolecular cyclic adduct, as described in Scheme 7, was formed when **Ru(T)-ODN(G)** was reacted alone or in the presence of non-complementary strands. Yet, when reacted in the presence of its complementary strand, and after 30 min illumination, an intermolecular photo-cross-linking process occurred between the TAP ligand of both **Ru(T)-ODN(G)** and **Ru(T)-ODN(T)** molecules and the guanine base of the complementary strand. No intramolecular process was detected in this case (Figure 17, Scheme 9) with no competition from the G nucleotide on the probe sequence is taking place. In addition, the intramolecular adduct, i.e., the reaction of the Ru complex with a G nucleotide in the probe sequence can be obtained, but it is occurring only in the absence of the target sequence. Therefore, this intramolecular adduct, named seppuku, prevents any side reaction from occurring [143].

This phenomenon was explained a few years later through a thorough biochemical analysis of the interaction of [Ru(TAP)_2_phen”]^2+^ with free guanine along with DNA oligonucleotides using molecular modelling simulations [144]. The formation of the intrastrand photoadduct requires a strong distortion of the oligonucleotide which cannot occur in the presence of the complementary strand containing a reachable guanine base, leading to a 100% interstrand photo-cross-linking process. Thereby, the formation of the intramolecular photoadduct enables the prevention of side reactions in the absence of the complementary strand or in the presence of non-complementary targets, without competing with the intrastrand process in the presence of the complementary strand. These results show the interest of ruthenium-based oligonucleotides for gene silencing methods but also as a way to prevent and detect mismatches in vivo. 

In light of these results, [Ru(TAP)_2_phen”]^2+^ was tested in 2013 as a therapeutic agent against cervical cancer [145] through an antisense-based strategy. Cervical cancer is associated to human papillomavirus (HPV) infection [146]. The HPV oncogene E6 is able to form an inhibiting complex with the p53 nuclear phosphoprotein [147,148]. The latter, in its primal state, possesses antiproliferative properties by promoting cell cycle arrest and/or leading stressed cells to apoptosis [149]. The complexation of p53 by E6 leads to the abrogation of p53 functions, which cause the proliferation of cancer cells and tumour progression. Hence, antisense oligonucleotides targeting E6 could lead to a knockdown of this HPV oncogene and concomitantly restore the antiproliferative role of the p53 protein. In this context, [Ru(TAP)_2_phen”]^2+^ was first coupled to an oligonucleotide probe containing a guanine base and targeting a specific sequence of E6 at position 324 of the gene. 

**Figure 18:**
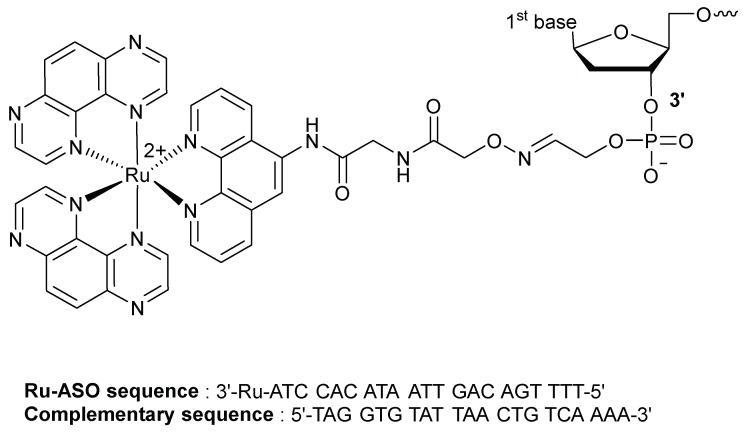
Structure of **Ru-ASO** and its complementary sequence [145].

PAGE experiments showed that the ruthenium antisense oligonucleotide (**Ru-ASO**, Figure 18) in the absence of its complementary strand or with a non-complementary strand under irradiation gave, as expected, a cyclic adduct in 80% yield coming from an intramolecular reaction. Similarly, in the presence of its intended target, a photo-cross-linking process occurred also in 80% yield. An in vitro experiment with SiHa human cervical cancer cells revealed that **Ru-ASO** was able to inhibit, with specificity, the cell growth under light irradiation (45–50% of inhibition 24 h after irradiation). Western blot experiments attested that the inhibition was due to the decrease of E6 expression. The results showed that **Ru-ASO** reduces E6 expression with 60% efficiency, 24 h after irradiation while confocal microscopy studies revealed a restoration of p53 expression in these cells. Overall, this study demonstrated the efficiency and specificity of gene silencing therapy and gave preliminary results for the use of **Ru-ASO** in phototherapy. 

## 5. Enzymatic Incorporation of Ru Complexes into DNA

**Figure 19:**
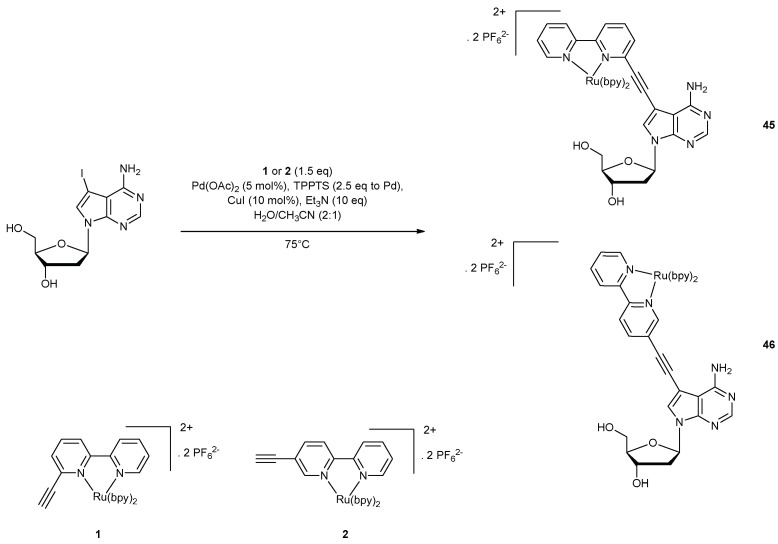
Synthetic pathway for the preparation of nucleoside triphosphates **45**, **46** [160].

The study of ruthenium complexes and more specifically of [Ru(bpy)_3_]^2+^ (bpy = 2,2’-bipyridine) [150] has shown that the photophysical properties of ruthenium complexes could be modulated by the nature of the ligand, making them good candidates as DNA probes. Thereby, in 2007, Hocek et al. took an interest in the synthesis of purine nucleosides (2’-deoxyadenosine [151] and 9-benzyladenine [152]) bearing in position 8 of the nucleobase phenanthroline or bipyridine ruthenium complexes. Their choice was motivated by the lack of reports concerning ruthenium probes conjugated to purines, in contrast with numerous reports of pyrimidine Ru-probes (vide supra). Unfortunately, due to the greater difficulties in preparation and chemical incorporation of this kind of molecules, the synthetic pathways developed gave low to moderate yields. As an alternative, Ru complexes were appended at position 8 of the nucleobase of purine triphosphates but these analogues revealed to be poor substrates for DNA polymerases [153,154]. As 7-substituted molecules were efficiently incorporated by the Pwo DNA polymerase [153,155,156,157], a new synthetic pathway was developed based on the Sonogashira reaction, to synthesise 7-deaza-2’-deoxyadenosines bearing bipyridine ligands and their ruthenium complexes. The complexes were connected to the nucleobase through an acetylene linker which ensured a certain rigidity often required for an efficient polymerase acceptance [158] as well as ease of synthesis. First, 7-iodo-7-deaza-2’-deoxyadenosine and two ruthenium-containing acetylenes (**45**, **46**) were prepared according to literature protocols [152,159]. The coupling was performed through a Sonogashira reaction in a water/acetonitrile mixture (Figure 19). The choice of this solvent was made in order to be applicable for a further labelling of nucleoside triphosphates. Interestingly, the reaction gave much higher yields compared to 8-substituted-2’-deoxyadenosine analogues (16% for the first while the second could not be isolated). 

**Scheme 10:**
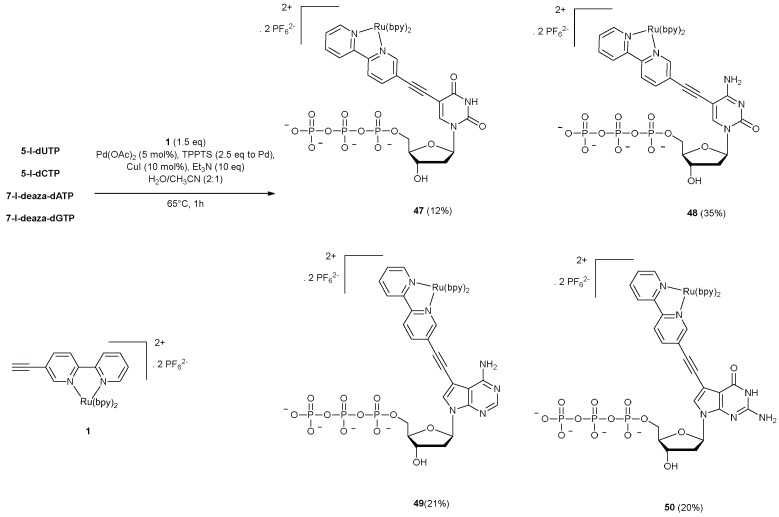
Synthetic pathway for the preparation of nucleotides **47**–**50** [163].

Photophysical and redox experiments showed a weak red luminescence with a low quantum yield for **45** and a moderate quantum yield of 0.03 for **46.** Both molecules gave electrochemical oxidation (1.2 V), showing preliminary results for redox labelling of DNA. The use of this Sonogashira reaction enabled the incorporation of various metal complexes (i.e., Os, Fe, Ni) [161,162] on the nucleobase to yield both modified nucleoside triphosphates and DNA oligonucleotides. In 2009, [Ru(bpy)_3_]^2+^ was successfully linked to nucleoside triphosphates **47**–**50** (Scheme 10) [163]. The coupling reactions were conducted on the known analogues 5-iodo-2’-deoxyuridine 5′-triphosphate (**5-I-dUTP**), 5-iodo-2’-deoxycytidine 5′-triphosphate (**5-I-dCTP**), 7-iodo-7-deaza-2’-deoxyadenosine 5′-triphosphate (**7-I-deaza-dATP**), and 7-iodo-7-deaza-2’-deoxyguanosine 5′-triphosphate (**7-I-deaza-dGTP**) [154,164,165,166,167]. The Sonogashira reaction was ended in 1 h, even if total conversion was not achieved because of the partial decomposition and hydrolysis of the starting and final triphosphates. The authors also mentioned the quick hydrolysis (a few weeks) of the final products in water at −20 °C. 

After HPLC purification, the efficiency of the polymerase incorporation of the modified triphosphates **47**–**50** was tested. Only Vent (*exo*^−^) and Pwo of the four polymerases that were tested gave full-length products under primer extension (PEX) reaction conditions. Other PEX experiments showed the successful incorporation of these modified dNTPs into diverse DNA sequences. However, the incorporation of two modified nucleoside triphosphates at adjacent positions led to an early termination of the polymerisation reaction. 

Electrochemical studies were then carried out to ensure the potential of the modified dNTPs for DNA labelling. Unfortunately, the potential of the ruthenium (1.1–1.25 V) is overlapped by the potential of guanine bases (1.1 V), making **47**–**50** rather poor candidates for redox labelling of DNA. The luminescence of ruthenium complexes still allows the use of these molecules for luminescent spectroscopy detection.

## 6. Conclusions

Chemical modification of oligonucleotides is an alluring strategy to improve the potency of DNA and RNA to serve in a number of practical applications. For instance, the inclusion of modifications on the sugar and/or phosphate backbone of nucleotides increases the resistance of antisense oligonucleotides to nuclease degradation [116,168], the incorporation of fluorescent or electrochemical tags enables the localisation of biological targets both in vitro and in vivo [169,170], or the appendage of artificial nucleotides facilitates the immobilisation of oligonucleotides on solid supports [171,172]. The two main strategies for the synthesis of modified oligonucleotides are DNA automated synthesis and the polymerisation of modified nucleoside triphosphates. In this review, we describe several approaches for the covalent linkage of ruthenium complexes on various locations of DNA oligonucleotides. 

The covalent linkage between nucleic acid derivatives and ruthenium complexes allows the direct application of the inherent properties of ruthenium complexes to probe DNA, detect mismatches in vivo, serve as gene silencing agents, or to stabilise DNA structures with the intercalation of ruthenium complexes. The elucidation of different mechanisms involved in DNA has also been possible, with the development of light switches, probes for the study of thermal stability or kinetics in DNA or the elucidation of several electron transfer mechanisms. The ease of functionalization of ruthenium complexes has allowed the incorporation of two different oligonucleotides for the elaboration of complex nanostructures. Depending on the coordination number of ruthenium, different shapes could be created and, among them, three-way DNA structures. As three-branched DNA structures are often found in the process of replication or recombination, this work could contribute to the better understanding of these biological processes.

Yet, among the numerous applications described in this review and to our surprise, few studies have been carried out to link the anticancer potency of ruthenium complexes to oligonucleotides. However, we are confident that more remarkable discoveries will be reported in the near future, highlighting the full potential of these compounds.

## Figures and Tables

**Table 1:**
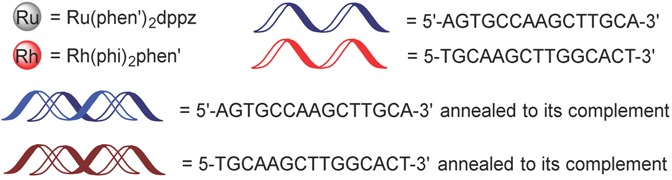
Luminescent intensity of intramolecular metal-DNA complexes relative to that of 5′-Ru(phen’)_2_dppz-AGRGCCAAGCTTGCA-3′, integrated from 500 to 800 nm [74].

Sample	Relative Intensity
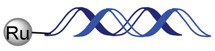	1.00
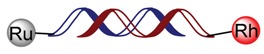	0.00
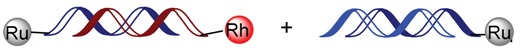	1.15
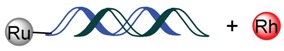	0.08
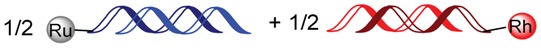	0.43

**Table 2:**
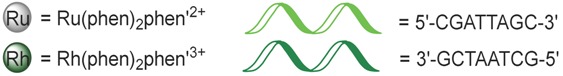
Luminescent lifetimes of tris(phenanthroline)metal-DNA complexes. Samples were dissolved in buffer (5 mM tris, 50 mM NaCl, pH 7.2) to ≤5 μM and experiments performed at 10 °C to ensure duplex stability [74].

Sample	Lifetime (ns)
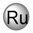	820
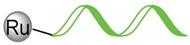	820
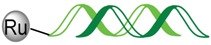	830
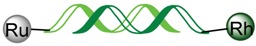	830

**Table 3:** Table showing the position of the osmium acceptor, [Os(bpy)_2_(3-ethynyl-1,10-phenanthroline)]^2+^, in various 19-mer duplexes where the ruthenium donor position, [Ru(bpy)_2_(3-ethynyl-1,10-phenanthroline)]^2+^, remains the same (U-19). The duplex containing strand 1 and 2 is used as a control.

Strand	Sequence	Os Position	Weighted Average Time (μs)
1 2	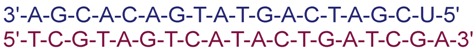		
3 2	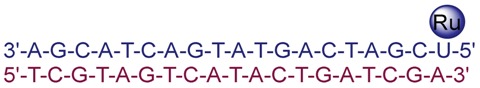		1.31 ± 0.01
3 4	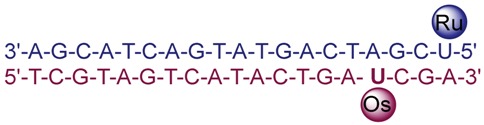	U-4	0.20 ± 0.06
3 5	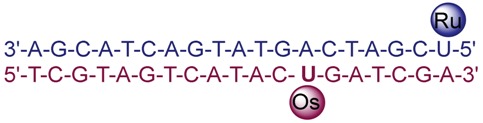	U-7	0.27 ± 0.01
3 6	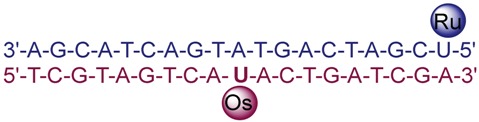	U-10	0.63 ± 0.01
3 7	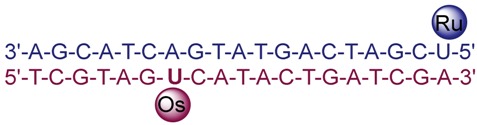	U-13	1.02 ± 0.12
3 8	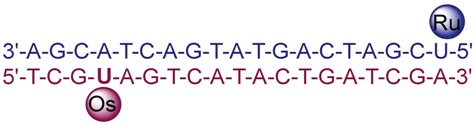	U-16	1.24 ± 0.02
3 9	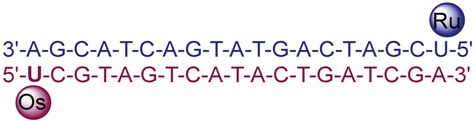	U-19	1.27 ± 0.01

**Table 4:** Showing the position of the osmium acceptor, [Os(bpy)_2_(3-ethynyl-1,10-phenanthroline)]^2+^, in various 19-mer duplexes where the ruthenium donor position, 1, remains the same (U-19) [92].

Strand	Sequence	Os Position	Weighted Average Time (μs)
10 2	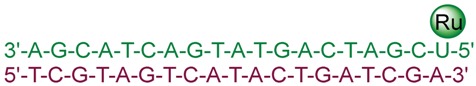		0.59 ± 0.01
10 4	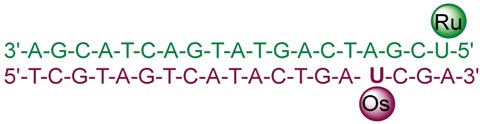	U-4	0.07 ± 0.01
10 5	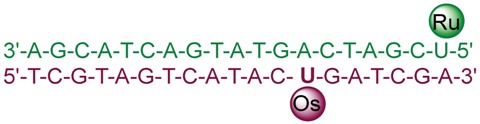	U-7	0.18 ± 0.01
10 6	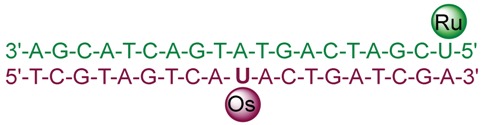	U-10	0.37 ± 0.01
10 7	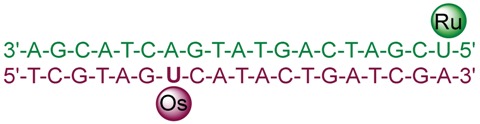	U-13	0.52 ± 0.01
10 8	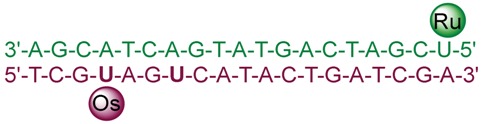	U-16	0.58 ± 0.01
10 9	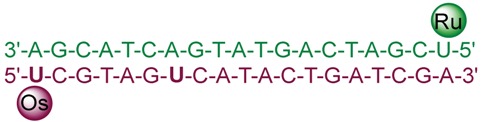	U-19	0.59 ± 0.01

## Abbreviations

The following abbreviations were used in the manuscript:

Bpy	2,2’-Bipyridine
DMAP	4-Dimethylaminopyrimidine
DMF	Dimethylformamide
DMSO	Dimethylsulfoxide
dNTP	Deoxyribose nucleoside triphosphate
Dppz	Dipyrido[3,2:a-2′,3′:c]phenazine
Im	Imidazole
LMCT	Ligand-to-metal charge transfer
Me2dppz	9,10-Dimethyl-dipyridophenazine
MLCT	Metal-to-ligand charge transfer
ODN	OligoDeoxyriboNucleotide
Oligo	Oligonucleotide
PAGE	Polyacrylamide gel electrophoresis
Phen	1,10-Phenanthroline
Phen’	5-(Glutaric acid monoamide)-1,10-phenanthroline
PNA	Peptide nucleic acid
Py	Pyridine
TAP	1,4,5,8-Tetraazaphenanthrene
Terpy	Terpyridine
TFA	Trifluoroacetic acid
TPAC	Tetrapyridoacridine
TPPTS	Triphenylphosphine-3,3′,3′′-trisulfonic acid trisodium salt
